# Effect of Heat Treatment on Yellow Field Pea (*Pisum sativum*) Protein Concentrate Coupled with Membrane Ultrafiltration on Emulsification Properties of the Isolated >50 kDa Proteins

**DOI:** 10.3390/membranes13090767

**Published:** 2023-08-30

**Authors:** Nancy D. Asen, Rotimi E. Aluko

**Affiliations:** 1 Department of Food and Human Nutritional Sciences, University of Manitoba, Winnipeg, MB R3T 2N2, Canada; asennda@myumanitoba.ca; 2 Richardson Center for Food Technology and Research, University of Manitoba, Winnipeg, MB R3T 2N2, Canada

**Keywords:** emulsions, membrane ultrafiltration, protein aggregates, interfacial activity, pea protein, heat

## Abstract

The aim of this paper was to determine the emulsification properties of protein aggregates obtained from heat pretreated yellow field pea protein concentrate (PPC). PPC dispersions were prepared in distilled water (adjusted to pH 3.0, 5.0, 7.0, or 9.0), heated in a water bath (100 °C) for 30 min, centrifuged and the supernatant passed first through a 30 kDa membrane and, then, the first retentate (>30 kDa) through a 50 kDa membrane. The 50 kDa membrane separation yielded a second retentate (>50 kDa proteins), which was isolated for emulsification studies. The near UV circular dichroic spectra of the protein samples showed more unfolded structures at pH 3.0 and 5.0 than at pH 7.0 and 9.0. The presence of small and spherical oil droplets of emulsions stabilized by the >50 kDa proteins at pH 3.0, 7.0, and 9.0 was confirmed by confocal laser scanning microscopy images. Emulsions stabilized at pH 7.0 and 9.0 had a narrower size distribution range than at pH 3.0 and 5.0. A narrow oil droplet size distribution range and lower interfacial protein concentrations of the emulsions stabilized by the >50 kDa proteins were observed at the corresponding pH of the heat treatment when compared to other pH values. Emulsions stabilized by the >50 kDa proteins exhibited a relatively low flocculation and coalescence index, which infers relative stability. The results from this work suggest that heat pretreatment of the PPC led to the formation of new protein aggregates, especially FT9 with enhanced emulsification properties, at some of the test conditions when compared to the unheated PPC.

## 1. Introduction

Emulsions are commonly used in food formulations (e.g., milk, yoghurt, salad dressing, desserts, toppings, and marshmallows) and have been more recently used in encapsulation technology for the delivery of nutrients and nutraceuticals. The main feature of a food emulsion is the presence of two immiscible liquid phases separated by a thin layer (interface), where one phase is dispersed into another as droplets after agitation using an external force [1,2,3,4]. Molecular incompatibility between the two phases necessitates a phase separation over time and this occurrence is caused by several interrelated factors like creaming, sedimentation, flocculation, and coalescence. The stability of emulsions is essential in food formulations and can be achieved by providing structures that will withstand changes over time during the storage period. Emulsifiers, or surface-active ingredients (interfacial layer modifiers) and texture modifiers (increase the viscosity of the continuous phase) are commonly used to form and stabilize emulsions and ensure kinetic stability.

Studies have shown that high molecular weight emulsifiers can stabilize emulsions by the formation of rigid interfacial films and conformations that increase the presence of steric repulsive forces [3,5,6]. Plant proteins are natural high molecular weight emulsifiers, and this ability is conferred by the presence of different classes of amino acids (amphiphilicity), in addition to electrostatic (repulsive and attractive) and steric forces. The macromolecular size of food proteins acts as a stabilizer by increasing the viscosity of the continuous phase and providing steric hindrance against oil droplet coalescence [5,7]. Plant proteins can lower the interfacial surface tension through a high surface charge and high solubility in the continuous phase at pH values above the isoelectric point (pI), while strong interfacial viscoelastic films are favored by a low surface charge, high surface hydrophobicity, and high solubility at pH values below the pI [5,7,8]. 

The yellow field pea protein (*Pisum sativum* L.) has gained attention in food applications due to sustainability factors (i.e., agriculture, environment, health, versatility, low cost, and availability), as well as its techno-functional and physicochemical properties [9]. Studies have shown that pea protein can perform well as an emulsifier at levels that are comparable to other legumes, like lentils and faba beans in oil-in-water (O/W) emulsion systems [10]. However, like other plant proteins, isolated pea proteins consist mainly of storage proteins with a compact (highly folded) conformation that could impair emulsification due to poor flexibility and solubility. This is because a good emulsifier requires flexibility to unfold at the interface, rearrange and expose the hidden hydrophobic groups in the oil phase and the hydrophilic groups in the water phase [11].

Physical treatment techniques like heat and ultrafiltration (UF) have been used in the processing of protein ingredients with superior functionalities [5,12,13,14]. These treatments are relatively cheap and safe, thereby improving consumer acceptability of the protein ingredients because they are free of chemical or enzymatic modification and toxic residues. Specifically, conventional heat treatment (e.g., boiling, roasting, frying, and baking) is a standard processing method for many foods and, thus, the effect of elevated temperatures on the ingredients and systems is important. The hidden reactive groups (sulfhydryl and hydrophobic side chains) in an unfolded protein are exposed and their functionality is enhanced after heating [11]. The literature is replete with studies that have been carried out on emulsion and interfacial properties of food protein emulsifiers, such as modified soybean [6], milk [15], and pea protein at the O/W interfacial layer [16], as well as the effect of heat treatment on the functional properties of pea protein at different pH values [5,17]. Similarly, membrane ultrafiltration has been used to extract, fractionate, and concentrate protein solutions to produce ingredients with superior emulsification properties and protein contents [18,19]. Thus, the hybrid use of heat treatment and membrane UF at different pH values could enhance the emulsification properties of pea protein concentrate via improved mobility and the isolation of size-defined protein aggregates with distinct structural properties. 

Heating pea proteins up to 90 °C can produce protein aggregates with similar solubility as the unheated protein, but having enhanced emulsion capacity at pH 7.0 [11]. Similarly, pea proteins heated at 95 °C for 30 min showed increased creaming stability and protein adsorption at the O/W interface than the unheated protein [5]. This is because when proteins are heated slightly above the denaturation temperature, reversible structural unfolding occurs, and an intermediate molten globule state is formed [11]. Furthermore, heat treatment at elevated temperatures for longer periods produces heat stable, flexible and soluble polypeptides, due to an increase in hydration and solvation of the protein molecules [20,21]. Soluble aggregates are formed when proteins are heated at higher temperatures and for longer periods (e.g., at 80–100 °C for 15–30 min) to break non-covalent and/or disulfide linkages within neonatal agglomerates [22]. Ultrafiltration is a non-thermal process driven by pressure and membrane-based protein fractionation, concentration, desalting, and clarification to preserve the functionalities of isolated proteins [17]. Membrane ultrafiltration/diafiltration is the use of semi-permeable membranes to separate species in aqueous solutions, based on size, charge, or shape, through a high pressure and convection system [18,19,23].

In this work, we collected and freeze-dried >50 kDa fractions of soluble pea proteins obtained after the heat treatment of a commercial pea protein concentrate at 100 °C and different pH values. Based on the foregoing, the aim of this study was to use heat and ultrafiltration to produce protein ingredients with enhanced emulsification properties and strong interfacial protein films. 

## 2. Materials and Methods

### 2.1. Materials

Yellow field pea protein concentrate (PPC) was purchased from Nutri-Pea Limited (Portage La Prairie, MB, Canada). Biomax polyethersulfone ultrafiltration membrane discs (76 mm diameter; nominal molecular weight limit 30 and 50 kDa) were purchased from Sigma-Aldrich (St. Louis, MO, USA). All the reagents and chemicals used for analysis were of high purity, analytical grade and purchased from Sigma-Aldrich (St. Louis, MO, USA) or Fisher Scientific Company (Oakville, ON, Canada). Double distilled water was used in the preparation of the chemicals and reagents. 

### 2.2. Methods

#### 2.2.1. Membrane Ultrafiltration of Heat-Treated Pea Protein Concentrate

**Figure 1:**
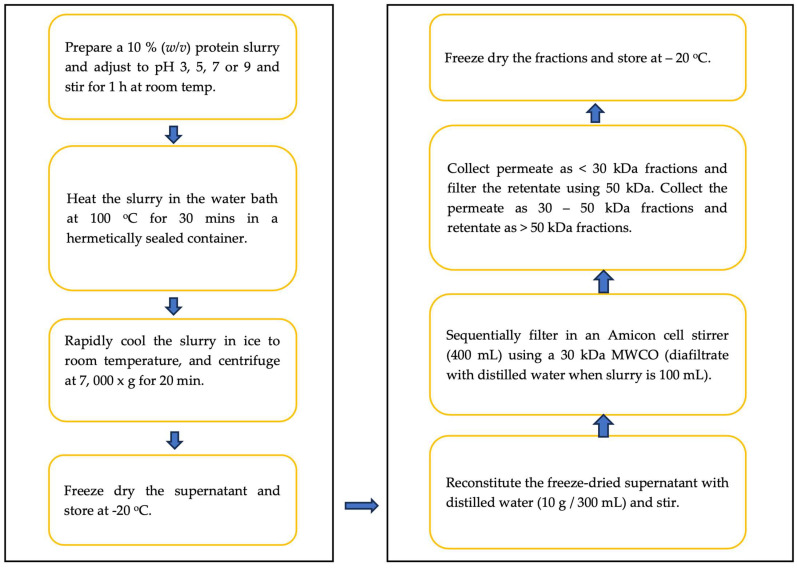
Configuration diagram for the sample preparation and ultrafiltration.

The protein aggregates and fractions were prepared as described by Asen and Aluko [17], and as shown in Figure 1. Mixtures of 10% (*w*/*v*) dispersion of the PPC (68.6% protein content) were prepared in distilled water, adjusted to pH 3.0, 5.0, 7.0, or 9.0, sealed, and heated in a water bath at 100 °C for 30 min. The mixtures were subsequently cooled to 20 °C, centrifuged at 7000× *g*, the supernatants collected and freeze dried. Subsequently, 400 mL of the freeze-dried supernatants were reconstituted in distilled water (10 g/300 mL) and each, sequentially, passed through 30 and 50 kDa membranes (filtration area = 41.8 cm^2^) using the Amicon^®^ 8400 stirred cell (Merck KGaA, Darmstadt, Germany), a high-pressure system driven by nitrogen gas at 75 psi (concentration mode). The cells were continuously stirred with a magnetic stirrer for 8 h, coupled with discontinuous diafiltration (once) when the sample volume dropped to 100 mL, to improve the flux and minimize membrane fouling. Three fractions (<30, 30–50, and >50 kDa) were collected for each pH treatment but based on the high protein content (>60%) and yield, the >50 kDa fractions were selected for analysis. The samples were labeled as fractionated treatments (FT), based on the pH at which the heat treatment was conducted: pH 3.0 (FT3), 5.0 (FT5), 7.0 (FT7), and 9.0 (FT9). The >50 kDa fractions were freeze dried and stored at −20 °C. The PPC was used as a control because subjecting it to similar conditions as the samples, but without the heat pretreatment, produced soluble protein yields that were too low and non-feasible for the studies carried out in this work. 

#### 2.2.2. Circular Dichroism Spectra Analysis

The near UV (250–340 nm) spectra of the isolated >50 kDa proteins were determined, as described by Aderinola et al. [24], at 25 °C using a J-815 spectropolarimeter (JASCO Corporation, Tokyo, Japan). The protein stock samples were prepared in 0.1 M acetate (pH 3.0–5.0), phosphate (pH 6.0–7.0), and Tris-HCl (pH 8.0 and 9.0) buffers and centrifuged (16,000× *g*; 30 min; 25 °C) to collect supernatants, which were then diluted to 4 mg/mL for the CD measurement. The spectra were determined using quartz cells with a path length of 1 mm and obtained as the average of three consecutive scans with automatic subtraction of the respective buffer spectra.

#### 2.2.3. Microstructure of Emulsion

The microstructure of the fresh emulsions was determined using a Zeiss confocal laser LSM 700 (CLSM) with a Zeiss Imager M2 microscope, according to a previously described method [25]. The emulsion samples prepared at 10 mg/mL protein concentration were stained with a mixture of Nile red and 0.1% (*w*/*v*) fluorescein isothiocyanate (FITC), which was dissolved in ethanol. The images were detected and analyzed at the wavelengths of 488 and 561 nm for Nile Red and FITC, respectively, using the Zen 3.4 (Zen Lite) software. 

#### 2.2.4. Droplet Size Distribution

The O/W emulsions were prepared as described by Adebiyi and Aluko [26], with some modifications. The emulsions were prepared using three sample concentrations (10, 15, and 20 mg/mL) in 5 mL of 0.1 M phosphate buffer at pH 3.0, 5.0, 7.0, or 9.0-, and 1 mL pure canola oil. Each mixture was homogenized at 20,000 rpm for 1 min using the 20 mm shaft on a Polytron^®^ PT 3100 homogenizer (Kinematica, AG, Switzerland). The droplet size distribution of the emulsions was determined in a Mastersizer 2000 (Malvern Instruments Ltd., Malvern, UK), with distilled water as a dispersant. 

#### 2.2.5. Flocculation (FI) and Coalescence Indices (CI)

The analyses were carried out using methods described by Castellani et al. [27]. The freshly prepared emulsions were diluted in either deionized water or 1% (*w*/*v*) sodium dodecyl sulfate (SDS), and the volume mean droplet diameter (d_4,3_) of the droplets was determined at 0 h and 24 h, using the Mastersizer 2000 protocols described above. The FI percentage was calculated at 0 and 24 h with the following equation [28]:

(1) FI (%)=[(d4,3 in water)/(d4,3 in 1% SDS)−1]×100

The CI was determined after 24 h of quiescent storage at 4 °C, as described by Palazolo et al. [29], as follows:

(2) CI (%)=[d4,3(24 h)/d4,3(0 h)]−1]×100

where d_4,3_ (0 h) and d_4,3_ (24 h) are the d_4,3_ of the droplets in the emulsions that were freshly prepared or after storage for 24 h, respectively, and determined using 1% SDS as a diluent.

#### 2.2.6. Interfacial Protein Concentration (Г)

The analysis was performed as described by Liang and Tang [9], with some modifications. The freshly prepared emulsions were centrifuged at 16,000× *g* for 60 min at room temperature. The cream layer (top layer) was carefully removed using a syringe and the serum (bottom layer) was removed with a syringe and filtered through a 0.22 µm filter (Millipore Corp., Burlington, MA, USA). The protein content of the filtrate was determined using the modified Lowry method [30], to obtain the unadsorbed protein content. Briefly, 20–100 μg/mL dilutions of the filtered sera were prepared, and the protein content determined using bovine serum albumin (BSA) as the standard. The Г (mg/m^2^) was calculated as:

(3) Percentage adsorbed protein (AP)=(Co−Cf)∗100/Co

(4) $$Г\;(\mathrm{mg}/m^{2})=(Co-Cf)*d3,2/6ø$$

where Co is the initial protein concentration of the emulsion and Cf is the protein content of the filtered serum, as determined by the modified Lowry method. The d_3,2_ was determined by the Mastersizer 2000 (Malvern Instruments Ltd., Malvern, UK) using deionized water as the dispersant, and ø is the oil fraction contained in the emulsion (0.167).

#### 2.2.7. Statistical Analysis

The analyses were carried out in triplicate and the mean values subjected to a three-way analysis of variance (ANOVA) using IBM SPSS Statistics for Windows, version 26.0. The significant difference between the mean values was set at *p* < 0.05 and determined by Duncan’s multiple range test. 

## 3. Results and Discussion

### 3.1. Circular Dichroism (CD) Spectra

**Figure 2:**
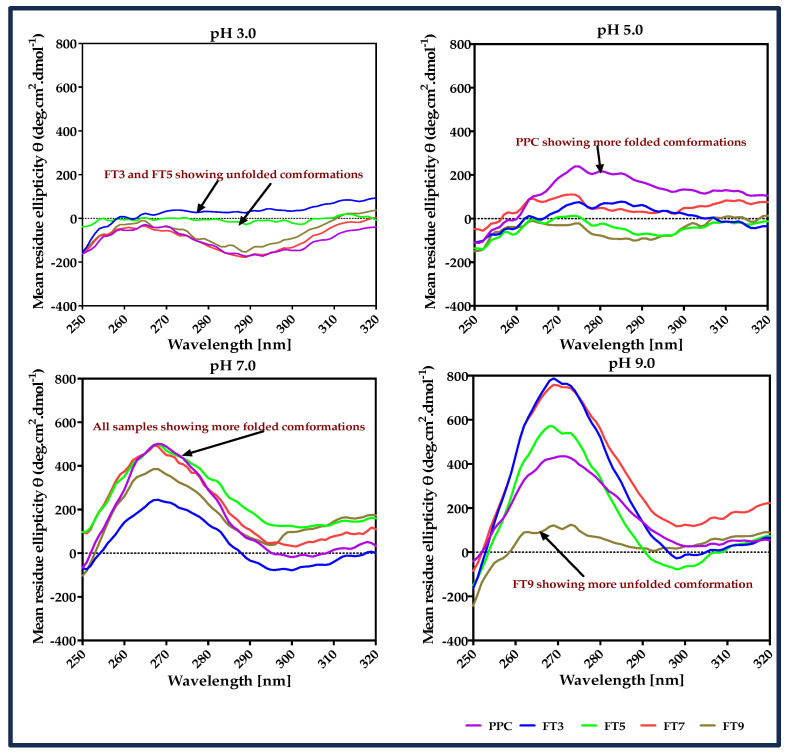
Near-UV circular dichroism spectra of pea protein concentrate (PPC) and isolated >50 kDa proteins. The PPC was heated (100 °C) at pH 3, pH 5, pH 7, and pH 9, followed by isolation of the >50 kDa proteins via membrane ultrafiltration to produce FT3, FT5, FT7, and FT9, respectively.

The structural changes in the protein ingredients during heat treatment were estimated indirectly by the absorption of light through the chromophores (Trp, Tyr, and Phe) and possibly the presence of disulfide linkages within the near UV (260–320 nm). This is important because the spatial conformation of an emulsifier influences efficiency and performance in the emulsification process. Characteristically, Trp shows a peak close to 290 nm with fine structure between 290–305 nm, Tyr has a peak between 275–282 with shoulders at longer wavelength obscured by Trp bands, while Phe has sharper but weaker bands between 255–270 nm and disulfide linkages at 250 nm [31,32]. The results showed that at pH 3.0 and 5.0, the >50 kDa proteins had relatively low ellipticity values, which indicate loose and disorganized structures when compared with the higher positive values at pH 7.0 and 9.0 (Figure 2). The main chromophore peak for FT3 and FT5 at pH 3.0 was Tyr (287 nm) and was in the hydrophilic environment because of the long wavelength. FT3 showed a typical Phe peak at 250 nm in the hydrophobic pocket or disulfide linkages, which is very similar to the observation with FT7, FT9, and PPC. On the other hand, FT5 had only a small peak for Phe, indicating a high degree of exposure to a hydrophilic environment due to structural disorganization [31], which is consistent with conformation associated with isolation at the pI. The results suggest that the more unfolded protein structure at low pH increased the distance between the hydrophobic groups. The major peak was Tyr at 289 nm for PPC, FT7 and FT9, which indicates exposure to the hydrophilic environment. A Trp transition occurred at 302 nm and was distinct as a small shoulder for PPC. Slightly higher dichroic values for PPC at pH 5.0 suggest a more compact and organized structure than the heat-treated samples at this pH and a small trough was seen at 252 nm. At pH 7.0 and 9.0, the protein samples exhibited an ordered structure with a Tyr peak between 267–268 nm in a hydrophobic environment and the high peak spectra, which depicts the presence of more ordered forms and increased interactions by the aromatic amino acids resulting from protein–protein interactions [33]. However, FT9 showed a loose structure at pH 9.0, as seen by the low dichroic values compared with the other samples and, apart from FT7, all the other fractions showed similar low dichroic values than PPC at the corresponding pH of the heat pretreatment, signifying unfolded structures with higher susceptibility to increased surface net charge due to the presence of exposed reactive groups. Similarly, Aderinola et al. [24] reported that increased net charge on a protein could facilitate the formation of less structurally organized structures. 

### 3.2. Microstructure of the Emulsions

**Figure 3:**
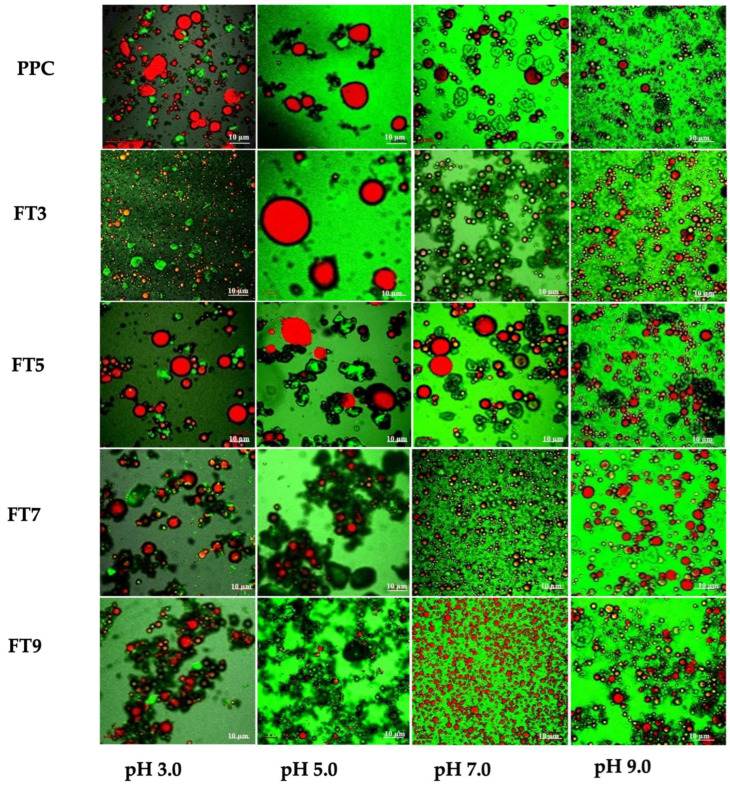
The confocal laser scanning microscopy (CLSM) images of the pea protein concentrate (PPC) and isolated >50 kDa proteins. The PPC was heated (100 °C) at pH 3, pH 5, pH 7, and pH 9 followed by isolation of the >50 kDa proteins via membrane ultrafiltration to produce FT3, FT5, FT7, and FT9, respectively. The emulsions were prepared with 10 mg/mL protein concentration at pH 3, 5, 7, and 9. The oil was stained with Nile red (red signals) and the protein with FITC (green signals). Bars = 10 µm.

The CLSM images of the O/W emulsions stabilized by PPC and the >50 kDa proteins were obtained using 10 mg/mL protein concentration at pH 3.0, 5.0, 7.0, and 9.0. The oil droplets are stained red with Nile red dye, while the proteins in the continuous phase are stained green with FITC. Thick layers are seen around the oil droplets for most of the emulsions and postulated to be the protein particles adsorbed to the interfacial layer in the emulsions (Figure 3). The images show that the different treatments produced emulsions with distinct morphological variations, dominated by inter-droplet attractive interactions between the oil droplets and multiple protein particles adsorbed to the interfacial layer, which could favor the emulsification properties [16,34]. All the samples showed signs of interactions at pH 3.0, 7.0, and 9.0, but at pH 5.0, the oil droplets showed more inter-particle distance, which could be due to increased insolubility of the proteins within the continuous phase to form [16]. The scanning electron microscope images (SEM) of the samples, as reported in our previous work [17], showed an amorphous floating mass and signs of networking and interaction within the structure of FT3, FT7, and FT9, while FT5 had a more discrete structure with lesser signs of interaction. Also, the droplets and aggregates at pH 7.0 and 9.0 were uniformly distributed, which could be a result of more efficient adsorption of the protein at the interfacial layer [35]. The spherical oil droplets in most of the protein fractions (except at pH 5.0) suggest better emulsion capacity of the aggregates than PPC-stabilized emulsions, which are seen as irregular droplets [36]. Most of the samples exhibited smaller oil droplets below or above pH 5.0, and numerous droplets at pH 7.0 and 9.0, which could reasonably infer superior emulsion formation capacity under those conditions. In particular, the FT7 and FT9 formed smaller and more uniformly distributed oil droplets at pH 7.0, which suggests a more efficient emulsification capacity than at other pH values and better than the PPC. As seen in Figure 2, the protein aggregates were highly unfolded at pH 3.0 and 5.0, and only moderately unfolded at pH 7.0 and 9.0. Moderate structural unfolding at higher pH could be an influencing factor on the efficiency of the emulsification process, leading to the ease of alignment of the hydrophobic groups at the interfacial layer and the droplets, while high unfolding at a low pH may have produced inflexible polypeptides. On the other hand, during the emulsification process, optimal unfolding of the >50 kDa proteins was achieved at the respective pH of the heat pretreatment, which will be further discussed in the following sections. The scanning electron microscope images (SEM) of the samples, as reported in our previous work [17], showed an amorphous floating mass and signs of networking and interaction within the structure of FT3, FT7, and FT9, while FT5 had a more discrete structure with lesser signs of interaction. Also, the droplets and aggregates at pH 7.0 and 9.0 were uniformly distributed, which could be a result of more efficient adsorption of the protein at the interfacial layer [35]. The spherical oil droplets in most of the protein fractions (except at pH 5.0) could suggest better emulsion capacity of the aggregates than PPC-stabilized emulsions, which are seen as irregular droplets [36]. Most of the samples exhibited smaller oil droplets below or above pH 5.0 and numerous droplets at pH 7.0 and 9.0, which could reasonably infer superior emulsion formation capacity under those conditions. In particular, the FT7 and FT9 formed smaller and more uniformly distributed oil droplets at pH 7.0, which suggests a more efficient emulsification capacity than at other pH values and better than the PPC. As seen in Figure 3, the >50 kDa proteins were highly unfolded at pH 3.0 and 5.0 and only moderately unfolded at pH 7.0 and 9.0. Moderate structural unfolding at higher pH could have been an influencing factor in the efficiency of the emulsification process, leading to the ease of alignment of the hydrophobic groups at the interfacial layer and the droplets, while high unfolding at low pH may have obstructed the process. On the other hand, during the emulsification process, optimal unfolding of the aggregates was achieved at their respective fractionation pH values, which will be seen as the results are further discussed in subsequent sections below. 

### 3.3. Macrostructure of the Oil-in-Water Emulsions at Different pH and 10 mg/mL Protein Concentration

**Figure 4:**
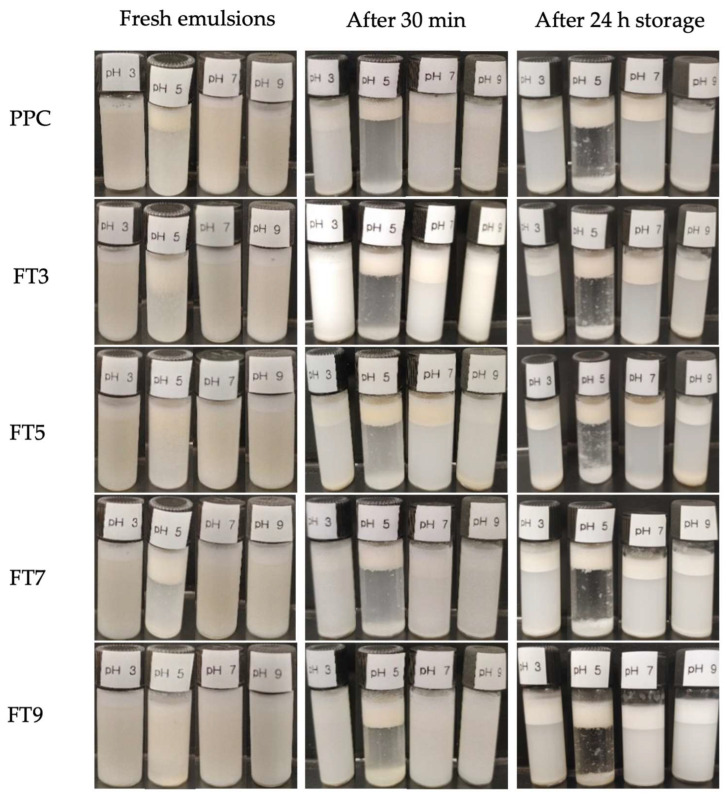
Digital images of oil-in-water emulsions prepared from the pea protein concentrate (PPC) and isolated >50 kDa proteins at 0 min, 30 min, and 24 h, with canola oil at 10 mg/mL protein concentration and pH 3.0, 5.0, 7.0, and 9.0. The PPC was heated (100 °C) at pH 3, pH 5, pH 7, and pH 9, followed by isolation of the >50 kDa proteins by membrane ultrafiltration to produce FT3, FT5, FT7, and FT9, respectively.

The macrostructures of the emulsions at 10 mg/mL protein concentration, in Figure 4, show that the emulsion properties were dependent on the pH. This observation corresponds with the pH related images of the droplets, as seen in Figure 3. The fresh emulsions (after 0 min) at pH 3.0, 7.0, and 9.0 had slight visible interface height through the volume of the emulsions, especially for FT9 and FT7 at pH 7.0. A similar observation was recorded for FT5 at pH 5.0 and pH 7.0 and, apart from FT9 at pH 5.0, the >50 kDa proteins and PPC showed some phase separation in the fresh emulsions at pH 5.0, which became more evident after storage for 30 min. The volume of the emulsified phase decreased for all the samples, except FT9 at pH 3.0, 7.0, and 9.0. After storage for 24 h, the interface height of the emulsions was ~30% of the total emulsion volume, and treatments at pH 5.0 showed a transparent continuous phase, while the emulsions prepared at the other pH values were cloudy due to partial phase separation. 

### 3.4. Particle Size Distribution

**Figure 5:**
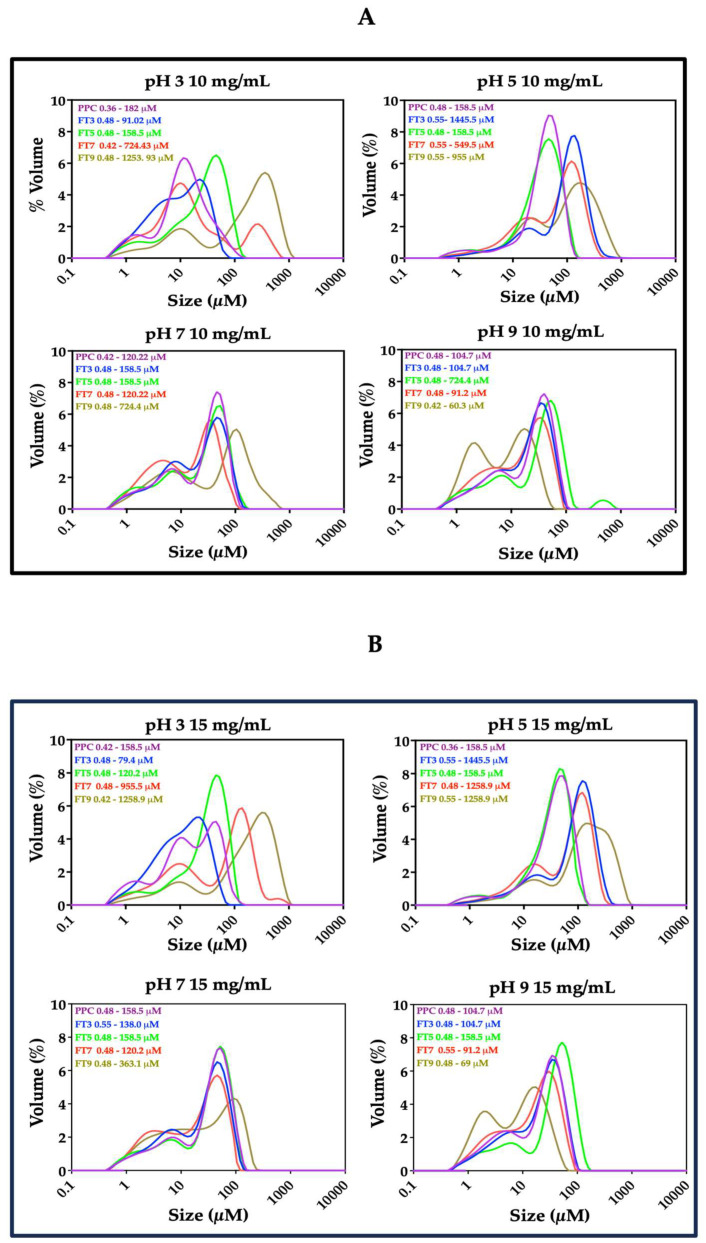

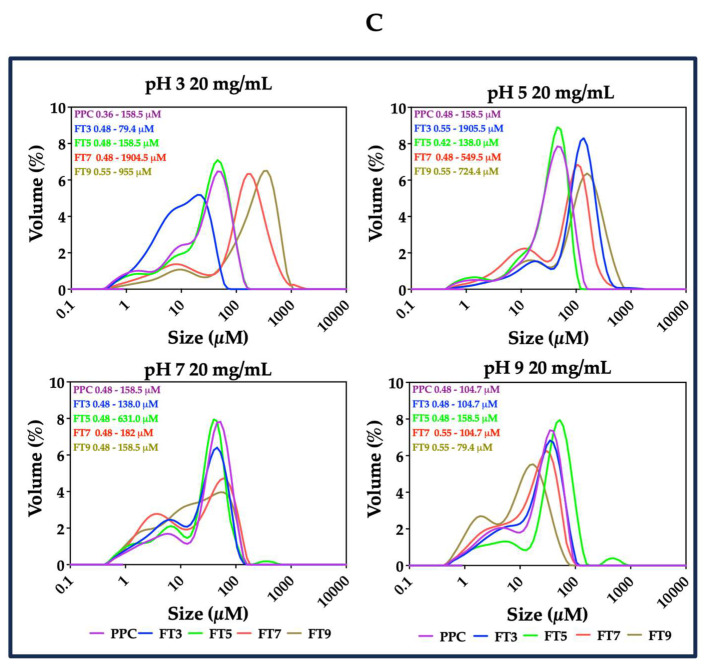
Oil droplet size distribution in the emulsions stabilized by pea protein concentrate (PPC) and isolated >50 kDa proteins at (**A**) 10 mg/mL, (**B**) 15 mg/mL, and (**C**) 20 mg/mL. The emulsions were prepared at different pH values and protein concentrations. The PPC was heated (100 °C) at pH 3, pH 5, pH 7, and pH 9, followed by isolation of the >50 kDa proteins via membrane ultrafiltration to produce FT3, FT5, FT7, and FT9, respectively.

Food emulsions are polydisperse (monomodal, bimodal, or multimodal) and are characterized based on the particle size distribution, by defining the dispersed phase into different classes [37]. Furthermore, the particle size distribution of protein-stabilized emulsions has a positive correlation with surface tension at the O/W interfacial layer [7]. Narrow particle size ranges could lower the surface tension and influence enhanced emulsification properties better than wide ranges. As shown in Figure 5, emulsions stabilized by PPC and the >50 kDa proteins exhibited mostly multimodal distribution, which indicates polydispersity, but the magnitude and the location of the prominent peak was dependent on the pH and protein concentration [5]. At pH 7.0 and 9.0, the emulsions had maximum particle sizes close to 100 µm (d_3,2_ was <8 μm at 10 mg/mL), in contrast to 1000 µm for pH 3.0 and 5.0 (d_3,2_ was >8 μm at 10 mg/mL).

The particle size range of the emulsions was narrower at pH 7.0 and 9.0 than at pH 3.0 and 5.0, and the aggregates produced the narrowest oil droplet size range at their corresponding fractionation pH, which is postulated to be the reason for the enhanced emulsification properties under those conditions. The lowest oil droplet size range was achieved in emulsions stabilized by FT3 at pH 3.0 (0.48–91.02 μM) and FT9 at pH 9.0 (0.42–60.30 μM), both at 10 mg/mL. It is worth mentioning that emulsions stabilized by FT9 at pH 3, especially at 10 and 15 mg/mL, showed a wider oil droplet size range than FT9-stabilized emulsions at other pH values, and this is seen in Figure 3 as non-uniformly distributed particles. Also, wider particle size distributions in the emulsions stabilized by the protein ingredients at pH 3.0 and 5.0 could have been mostly related to the non-spherical and non-uniformly distributed particles, as seen in Figure 3. Understandably, the largest volume fractions for >10 µm sizes occurred mostly at pH 5.0, which indicates low emulsification capacity at pH close to the pI, and corresponds to the microstructures and macrostructures reported in Figure 3 and Figure 4, respectively. The results also relate to the pH-dependent solubility of the isolated protein aggregates, as reported in our previous study [17].

### 3.5. Interfacial Protein Concentration (Г)

The level of protein present at the interfacial layer is measured as the surface load, which is the amount of protein adsorbed per unit area of the interface (mg/m^2^). The surface load estimates the minimum emulsifier quantity required to cover all the droplets [37] and a lower value is an indicator of stronger efficiency of an emulsifier [38]. The amount of emulsifier needed to stabilize an emulsion depends on the surface load because the higher the Г values, the more emulsifier is required for stability [39]. The percentage protein adsorption to the surface of the droplets (Table 1) was generally higher at ≤pH 5.0 than at >pH 5.0 (with a few exceptions), which suggests an inverse relationship with the level of interfacial charges. This observation corresponds with the smaller droplet size in emulsions stabilized at high pH (Figure 3) and the narrow particle size distribution of emulsions stabilized at high pH values (Figure 5). A high level of adsorbed proteins was reported in whey protein microgels (WPM), which were screened out with salt, and only 50% adsorption occurred for the charged WPM [40]. The results in Table 2 show that all the protein samples had low Г values below and above the isoelectric point, and at 10 mg/mL protein concentration, which could infer relatively better emulsification properties corresponding to the microstructure and the oil droplet size distribution of the emulsions (Figure 3). Our previous study reported increased solubility (~35% for FT3 and FT9 vs. 15% for PPC) and surface charge (the highest was −20 mV for FT9 at pH 9.0) in the protein samples above their isoelectric point of pH 4.0–5.0 [17], which is consistent with the protein surface coverage reported in this work. The mean oil droplet size (d_3,2_) was smaller at below and above pH 5.0, the pI of pea proteins. This observation could be because small droplets have a large surface area, which would require less protein coverage at the interface, while a decrease in the surface area of large droplets would require more protein coverage per unit area [41]. Large droplets and a reduced charge (<pH 5.0) could lead to protein accumulation at the interfacial layer, while repulsive forces facilitated by charged particles will prevent accumulation [38,42]. The properties of most protein-stabilized emulsions are pH dependent because the net surface charge influences the surface tension and adsorption at the interfacial layer [7].

Furthermore, there was an increase in the Г values with increasing protein concentration for all the samples, which is postulated to be caused by the formation of multiple layers at the interface from the excess protein present, as observed with other globular proteins [37,41]. Excess protein leads to increased polymerization, but at low protein concentration, only few large particles can be formed through collision-induced aggregation [43,44]. A similar observation can be seen in Table 1, where the percentage of adsorbed protein for the samples at pH 7 and 9 was higher at 10 and 20 mg/mL than at 15 mg/mL, but the reason for the non-linear concentration dependency is not clear. However, it is possible that the decreased values from 10 to 15 mg/mL at some pH indicate increased solubility in the aqueous phase, which reduced the protein–oil interactions. When the protein concentration was increased to 20 mg/mL, there was a higher level of protein aggregation, which enhanced accumulation at the oil–water interface. Overall, Table 2 shows that the emulsions stabilized at pH 7.0 and 9.0 by the isolated >50 kDa proteins had slightly lower Г values than the PPC, which corresponds to a narrower range in oil droplet size as reported and relatively high stability towards flocculation. The isolated >50 kDa proteins had lower Г values than the PPC at low protein concentration and at the pH of the heat pretreatment, as earlier observed with the CD spectra for the protein ingredients (Figure 2), oil droplet size distribution (Figure 5), and emulsification activity index, reported in our current and previous studies [17], respectively. FT9 had high Г values at pH 3.0 and 5.0, which suggests poor emulsification capacity under those conditions, and this observation corresponds to the wider range in oil droplet size distribution at pH 3.0 and 5.0 (Figure 5) and the uneven distribution of oil droplet sizes, as revealed by the CLSM (Figure 3).

The Г values of PPC and isolated >50 kDa proteins exceeded 5 mg/m^2^ for emulsions stabilized at <pH 5.0, which could infer the formation of multiple layers of unfolded globular proteins on the interface [42]. Lower Г values (<4 mg/m^2^) may suggest the presence of a completely unfolded protein in the conformation of trains, loops, and tails [45], while higher Г values (>10 mg m^2^) are associated with biopolymeric emulsifiers [45,46]. In the current study, the Г values >10 mg m^2^ were only exhibited by FT9 at pH 3.0 and 5.0, and by FT3 at pH 5.0. The behavioral pattern of the proteins varies with the pH values due to the different interactions and the integrity of the interfacial layer, which is highly dependent on the concentration of the adsorbed protein.

### 3.6. Flocculation and Coalescence Index

**Figure 6:**
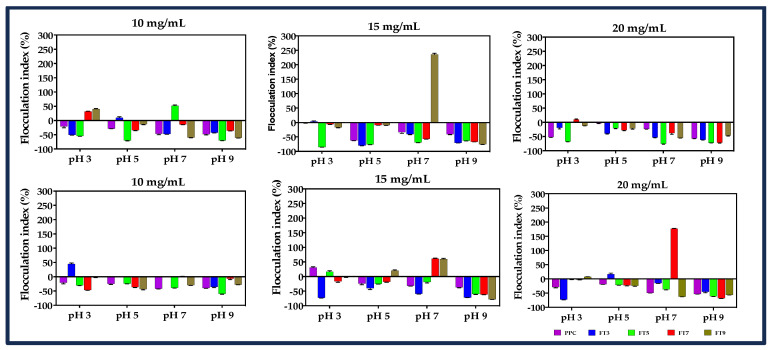
Percentage flocculation index (0, 24 h) of pea protein concentrate (PPC) and isolated >50 kDa proteins. The PPC was heated (100 °C) at pH 3, pH 5, pH 7, and pH 9, followed by isolation of the >50 kDa proteins by membrane ultrafiltration to produce FT3, FT5, FT7, and FT9, respectively. The emulsions were prepared at different pH values and protein concentrations.

The storage stability of emulsions can be predicted from the flocculation (flocs) and coalescence properties, as these factors cause emulsion breakdown over time [47]. Flocs are formed when two or more emulsified oil droplets stick together to form aggregates without losing their individual shapes, while coalescence occurs by merging two or more droplets to form a single larger droplet. Therefore, an increased level of flocculation reflects decreased emulsion stability. The flocculation index (FI) of freshly prepared and stored emulsions (24 h) was determined as relative changes in the d_4,3_ values, using water and 1% (*w*/*v*) SDS as dispersants. In Figure 6, the results show that the FI was dependent on the pH and protein concentration. At 0 h, stability against flocculation was achieved in the order pH 9.0 > pH 5.0 > pH 7.0 > pH 3.0 and the highest stability was achieved at 20 mg/mL protein concentration. After 24 h, the order changed slightly to pH 9.0 > pH 5.0 > pH 3.0 > pH 7.0 (*p* ≤ 0.05). Peng et al. [5] reported that the FI values of emulsions stabilized by heated pea protein increased with the increasing protein concentration from 0.1–0.3%, but a sharp decline occurred at >0.3% protein concentration. The dominance of inter-droplet attractive interactions at low protein concentration and interactions between adsorbed and unadsorbed protein at high protein concentration could predispose the emulsions to flocculation [9,16]. However, the formation of agglomerated oil droplets could influence emulsion stability by increasing the viscosity of the aqueous phase. Emulsions stabilized at pH 5.0 and 9.0 displayed slightly better stability against flocculation than the other treatments (*p* > 0.05). Stability at pH 5.0 could be due to the low protein solubility, which could have formed precipitated particles that provide physical separation of the oil droplets. In contrast, at pH 9.0, the strong electrostatic repulsion force between the interfacial membranes that surround the oil droplets coupled with steric repulsion, due to the size and solubility of the protein aggregates, could have contributed to better emulsion stability [25]. Furthermore, charged proteins could form a dense particle disc between the interface of neighboring droplets to maintain stability against flocculation and coalescence [40]. A decline in stability against flocculation occurred after storage for 24 h, and the FI values for treatments at pH 7.0 increased for all the protein concentrations, while emulsions prepared at pH 5.0 and 9.0 still maintained low FI index values.

**Figure 7:**
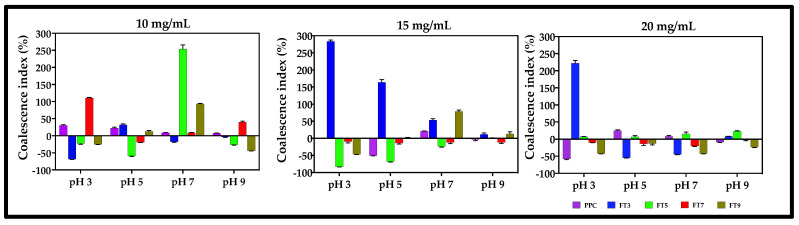
Percentage coalescence index of pea protein concentrate (PPC) and >50 kDa proteins. The PPC was heated (100 °C) at pH 3, pH 5, pH 7, and pH 9, followed by isolation of the >50 kDa proteins by membrane ultrafiltration to produce FT3, FT5, FT7, and FT9, respectively. The emulsions were prepared at different pH values and protein concentrations.

The coalescence stability of an emulsion is dependent on the integrity of the viscoelastic film formed at the interface [48]. The collapse of two or more small size oil droplets into one big size oil droplet reflects emulsion instability, hence a lower coalescence index means a more stable emulsion. The results showed that the sample type had more impact (*p* ≤ 0.05) on the coalescence index (CI) values than the treatment conditions of pH and protein concentration (Figure 7). FT3-stabilized emulsions had the least stability against the droplet coalescence (47%), while FT9-stabilized emulsions were more stable (~89%) than the PPC and the other fractions. This means that the viscoelastic film formed around the droplets of the FT3-stabilized emulsions depleted over time, probably due to weak cohesion between the polypeptide chains. Although the CI was not greatly influenced by the pH and the protein concentration (*p* > 0.05), emulsions stabilized at pH 5.0 and 9.0 maintained a slightly higher stability (~94%) against coalescence than the emulsions made at pH 3.0 and 7.0 (CI values 19 and 24%, respectively), which is consistent with the flocculation data. Better stability of the emulsions against coalescence was obtained at 20 mg/mL (<0%), whereas emulsions stabilized at 10 mg/mL protein concentration had a CI of 23%. The results suggest that a higher protein level enhanced the formation of more cohesive interfacial films with better resistance to coalescence. 

## 4. Conclusions

The CD spectra of the PPC and isolated >50 kDa proteins showed that the structures were less compact at pH 3.0 and 5.0 than at pH 7.0 and 9.0. This observation could be due to higher structural unfolding of the protein at lower pH during heat pretreatment, which was seen as low ellipticity values, whilst more moderate structural unfolding at high pH was seen as higher ellipticity values. This conformational change influenced the emulsification properties of the proteins because of the location of the hydrophobic groups (i.e., farther apart at pH 3.0 and 5.0, while closer together at pH 7.0 and 9.0). Interestingly, most of the isolated >50 kDa proteins (except FT7 at pH 7.0) had lower ellipticity values and formed emulsions with a narrower range of oil droplet sizes at the corresponding pH of the heat pretreatment than at other pH values. A similar observation was noted with the Г values of the emulsions, where the emulsions had the lowest values at the pH of the heat pretreatment. These indices suggest that the isolated >50 kDa protein fractions exhibited superior emulsion formation performance at the pH of the heat pretreatment, especially at low protein concentration, suggesting a more flexible structure, lower interfacial tension, and small particle size. However, while the emulsion quality of the >50 kDa proteins was dependent on the pH of the heat pretreatment, the stability against flocculation and coalescence were, in addition, also dependent on the protein concentration used to form the emulsions. The results indicate that heat-induced formation of high molecular weight (>50 kDa) proteins, especially FT9, could be used to enhance the emulsification properties of pea proteins. This is because the membrane fractionation of the unheated PPC did not yield >50 kDa proteins that were readily isolated from the heat pretreated PPC. It is, therefore, reasonable to conclude that the heat treatment of PPC led to the formation of the isolated >50 kDa proteins (probably aggregates), which are novel ingredients with potential for industrial use in the formulation of food emulsions. To the best of our knowledge, this work is the first to report on membrane isolation of heat-soluble proteins and their emulsification properties. This approach has the advantage of enabling the isolation of new protein structures created from heat treatment with properties that could facilitate the creation of new multi-phase food products. 

## 5. Limitations and Future Prospects of Study

This study characterized some emulsification indices of isolated >50 kDa yellow pea heat-soluble proteins. Heat-induced aggregation of globular proteins leads to the formation of primary and secondary aggregates with different morphologies, and each structure presents a unique functionality in food applications. Hence, the characterization and structure–function relationship of the aggregates would have provided additional information about the behavior of the soluble protein aggregates in food applications. Also, although most of the heat-induced aggregates exhibited superior functionality when compared with the PPC, additional treatment of the aggregates with other physical methods (i.e., microfluidization or conjugation) could improve their emulsification properties. 

To improve on the findings from the study so far, the next step would be to use a hybrid approach to maximize the structural modulation of yellow field pea protein for enhanced emulsification properties. Another proposition is the characterization and the structure–function relationship of the different protein aggregates and a comparative study with aggregates formed from milk protein under the same processing conditions. The translation of these ingredients into food products (i.e., beverages or baked goods) and the sensory evaluation of these products by consumers would reveal the acceptability and value of these novel ingredients.

## Figures and Tables

**Table 1:** Percentage of adsorbed protein for pea protein concentrate (PPC) and isolated >50 kDa proteins.

			^†^ Adsorbed Protein (%)		
* Sample	Protein Conc. (mg/mL)	pH 3	pH 5	pH 7	pH 9
PPC	10	98.30 ± 3.50 ^a^	97.30 ± 2.00 ^a^	97.94 ± 1.00 ^a^	97.60 ± 0.80 ^a^
	15	96.50 ± 3.50 ^a^	99.73 ± 1.68 ^a^	65.43 ± 1.42 ^e^	94.08 ± 2.02 ^b^
	20	98.20 ± 3.50 ^a^	96.80 ± 2.40 ^a^	76.35 ± 1.26 ^d^	81.75 ± 1.80 ^c^
FT3	10	86.85 ± 3.30 ^c^	97.60 ± 2.31 ^a^	92.04 ± 1.63 ^b^	82.83 ± 2.02 ^c^
	15	49.27 ± 0.48 ^e^	94.74 ± 2.19 ^b^	69.72 ± 2.29 ^e^	78.72 ± 3.61 ^d^
	20	66.09 ± 4.15 ^d^	82.39 ± 1.84 ^c^	60.15 ± 1.68 ^f^	99.46 ± 4.68 ^a^
FT5	10	98.20 ± 3.92 ^a^	98.60 ± 2.57 ^a^	98.10 ± 2.01 ^a^	96.30 ± 1.49 ^a^
	15	99.64 ± 4.20 ^a^	98.20 ± 3.10 ^a^	63.95 ± 2.31 ^e^	89.52 ± 2.59 ^c^
	20	91.48 ± 4.65 ^b^	96.90 ± 2.34 ^a^	81.97 ± 1.91 ^c^	79.22 ± 1.69 ^d^
FT7	10	97.50 ± 4.70 ^a^	97.30 ± 2.63 ^a^	93.91 ± 1.21 ^b^	99.97 ± 1.30 ^a^
	15	96.80 ± 4.55 ^a^	98.20 ± 2.28 ^a^	46.19 ± 2.33 ^h^	80.95 ± 4.52 ^c^
	20	97.90 ± 4.26 ^a^	86.64 ± 1.85 ^c^	55.62 ± 4.51 ^g^	59.39 ± 2.13 ^f^
FT9	10	98.30 ± 5.08 ^a^	98.70 ± 1.66 ^a^	77.18 ± 2.00 ^d^	69.16 ± 0.84 ^e^
	15	99.46 ± 4.68 ^a^	98.30 ± 2.90 ^a^	37.36 ± 2.48 ^i^	71.70 ± 4.70 ^d^
	20	97.60 ± 4.51 ^a^	98.10 ± 2.90 ^a^	68.40 ± 1.51 ^e^	80.82 ± 4.02 ^c^

^†^ Different letters indicate significant differences at the *p* ≤ 0.05 level for each column. Each value is the mean and standard deviation of triplicate determinations. * Commercial brand pea protein concentrate (PPC); fractions (FT3, FT5, FT7, and FT9). The PPC was heated (100 °C) at pH 3, pH 5, pH 7, and pH 9, followed by isolation of the >50 kDa proteins via membrane ultrafiltration to produce FT3, FT5, FT7, and FT9, respectively.

**Table 2:** Surface protein concentration (mg/m^2^) for pea protein concentrate (PPC) and isolated >50 kDa proteins.

			^†^ Surface Protein Concentration (mg/m^2^)		
* Sample	** Protein Conc. (mg/mL)	pH 3	pH 5	pH 7	pH 9
PPC	10	1.53 ± 0.00 ^f^	3.65 ± 0.00 ^g^	1.89 ± 0.02 ^c^	1.87 ± 0.01 ^d^
	15	2.38 ± 0.00 ^e^	4.82 ± 0.05 ^f^	3.61 ± 0.06 ^a^	2.64 ± 0.06 ^c^
	20	4.23 ± 0.00 ^c^	6.74 ± 0.02 ^d^	3.71 ± 0.06 ^a^	3.37 ± 0.07 ^b^
FT3	10	1.14 ± 0.03 ^f^	6.35 ± 0.01 ^d^	1.79 ± 0.03 ^c^	1.69 ± 0.04 ^d^
	15	1.17 ± 0.01 ^f^	7.94 ± 0.18 ^c^	2.23 ± 0.07 ^b^	2.24 ± 0.06 ^c^
	20	2.01 ± 0.04 ^e^	10.68 ± 0.24 ^b^	2.41 ± 0.07 ^b^	4.26 ± 0.09 ^a^
FT5	10	2.23 ± 0.00 ^e^	3.54 ± 0.00 ^g^	1.89 ± 0.09 ^c^	2.03 ± 0.00 ^c^
	15	3.59 ± 0.09 ^d^	4.84 ± 0.34 ^f^	3.13 ± 0.07 ^a^	3.10 ± 0.03 ^b^
	20	4.48 ± 0.14 ^c^	5.90 ± 0.00 ^e^	3.33 ± 0.08 ^a^	4.17 ± 0.08 ^a^
FT7	10	1.70 ± 0.00 ^f^	4.40 ± 0.00 ^f^	1.44 ± 0.02 ^c^	1.63 ± 0.00 ^d^
	15	3.47 ± 0.00 ^d^	6.60 ± 0.06 ^d^	1.20 ± 0.06 ^c^	1.99 ± 0.05 ^d^
	20	7.85 ± 0.09 ^b^	6.61 ± 0.14 ^d^	1.86 ± 0.15 ^c^	2.12 ± 0.08 ^c^
FT9	10	3.99 ± 0.00 ^d^	6.10 ± 0.00 ^d^	1.75 ± 0.04 ^c^	0.74 ± 0.01 ^e^
	15	7.50 ± 0.15 ^b^	11.11 ± 0.00 ^b^	1.04 ± 0.07 ^c^	1.11 ± 0.02 ^d^
	20	12.69 ± 0.00 ^a^	12.53 ± 0.00 ^a^	2.29 ± 0.05 ^b^	1.90 ± 0.04 ^d^

^†^ Different letters (a–g) indicate significant differences at the *p* ≤ 0.05 level for each pH value. Each value is the mean and standard deviation of triplicate determinations. * Commercial brand pea protein concentrate (PPC); fractions, FT3, FT5, FT7, and FT9, prepared at pH 3.0, 5.0, 7.0, and 9.0 respectively. ** Protein concentrations at 10, 15, and 20 mg/mL. The PPC was heated (100 °C) at pH 3, pH 5, pH 7, and pH 9, followed by isolation of the >50 kDa proteins (FT3, FT5, FT7, and FT9, respectively) by membrane ultrafiltration.

## Data Availability

The data are available from the corresponding author upon reasonable request.
